# The Proteomic Analysis of Maize Endosperm Protein Enriched by Phos-tag^tm^ Reveals the Phosphorylation of Brittle-2 Subunit of ADP-Glc Pyrophosphorylase in Starch Biosynthesis Process

**DOI:** 10.3390/ijms20040986

**Published:** 2019-02-24

**Authors:** Guowu Yu, Yanan Lv, Leiyang Shen, Yongbin Wang, Yun Qing, Nan Wu, Yangping Li, Huanhuan Huang, Na Zhang, Yinghong Liu, Yufeng Hu, Hanmei Liu, Junjie Zhang, Yubi Huang

**Affiliations:** 1 College of Agronomy, Sichuan Agricultural University, Huimin Road 211#, Wenjiang District, Chengdu 611130, Sichuan, China; 2002ygw@163.com (G.Y.); lvyn@shhrp.com (Y.L.); sly1832633910@163.com (L.S.); wwyybb007@163.com (Y.W.); 18328652715@163.com (Y.Q.); 15982862780@163.com (N.W.); yangpingli103@gmail.com (Y.L.); hh820423@163.com (H.H.); 13958@sicau.edu.cn (Y.H.); 2 College of Science, Sichuan Agricultural University, Huimin Road 211#, Wenjiang District, Chengdu 611130, Sichuan, China; 03118663@163.com; 3 Maize Research Institute of Sichuan Agricultural University, Huimin Road 211#, Wenjiang District, Chengdu 611130, Sichuan, China; sclydx@163.com; 4 College of Life Science, Sichuan Agricultural University, Xingkang Road 46#, Ya’an 625014, Sichuan, China; hanmeil@163.com (H.L.); junjiezh@163.com (J.Z.)

**Keywords:** maize, AGPase, phosphorylation, brittle-2, phos-tag^TM^

## Abstract

AGPase catalyzes a key rate-limiting step that converts ATP and Glc-1-p into ADP-glucose and diphosphate in maize starch biosynthesis. Previous studies suggest that AGPase is modulated by redox, thermal and allosteric regulation. However, the phosphorylation of AGPase is unclear in the kernel starch biosynthesis process. Phos-tag^TM^ technology is a novel method using phos-tag^TM^ agarose beads for separation, purification, and detection of phosphorylated proteins. Here we identified phos-tag^TM^ agarose binding proteins from maize endosperm. Results showed a total of 1733 proteins identified from 10,678 distinct peptides. Interestingly, a total of 21 unique peptides for AGPase sub-unit Brittle-2 (Bt2) were identified. Bt2 was demonstrated by immunoblot when enriched maize endosperm protein with phos-tag^TM^ agarose was in different pollination stages. In contrast, Bt2 would lose binding to phos-tag^TM^ when samples were treated with alkaline phosphatase (ALP). Furthermore, Bt2 could be detected by Pro-Q diamond staining specifically for phosphorylated protein. We further identified the phosphorylation sites of Bt2 at Ser^10^, Thr^451^, and Thr^462^ by iTRAQ. In addition, dephosphorylation of Bt2 decreased the activity of AGPase in the native gel assay through ALP treatment. Taking together, these results strongly suggest that the phosphorylation of AGPase may be a new model to regulate AGPase activity in the starch biosynthesis process.

## 1. Introduction

Maize is one of the three crops with the highest production in the world [1]. Maize starch, accounting for about 70% of maize kernels, is not only widely used as main energy source to support the majority of the world’s population, but is also used as a feedstock for the production of industrial material and bioethanol [2]. Kernel starch is a crucial factor in determining the yield and quality of maize. It is a main goal of breeders to increase starch content and improve the quality of starch in maize.

Maize is generally an excellent source of starch which is synthesized by four enzymes: ADP-Glc pyrophosphorylase (AGPase; EC 2.7. 7.27), starch synthase (SS; EC 2.4.1.21), starch branch enzyme (SBE; EC 2.4.1.18) and starch debranch enzyme (SDBE; EC 3.2.1.68) [3]. AGPase catalyzes the synthesis of ADP-glucose and diphosphate from ATP and Glc-1-p [4], and the product ADP-glucose serves as the activated glucosyl donor in α-1, 4-glucan synthesis [5,6]. This reaction is a key rate-limiting step in maize endosperm starch synthesis. AGPase widely exists in plant leaves and cereal endosperm in a heterotetrameric α2β2 format and is composed of two identical large subunits (Sh2) and two identical small subunits (Bt2) in maize endosperm [7,8].

Previous studies suggest that AGPase activity is mainly modulated by allosteric regulation, thermal inactivation and redox regulation [9,10,11,12]. Some small molecule effectors are thought to regulate the activity of AGPase in an allosteric regulation fashion, such as 3-phosphoglycerate (3-PGA) [13], inorganic phosphate (Pi) [14], and sugars [15]. 3-phosphoglycerate (3-PGA) and inorganic phosphate (Pi) are demonstrated as activator and inhibitor, respectively, in most cereals [16,17,18]. 3-PGA activates AGPase by increasing the affinity for substrate G-1-P, but Pi inhibits the activity of AGPase by reversing the effect of 3-PGA [19]. The heat lability of AGPase is another regulation mechanism [9]. AGPase from maize endosperm will lose 95% of activity when it is heated at 57 °C for 5 min [7]. The heat lability of AGPase leads to grain loss during hot weather. It is an aim of breeders to generate new maize varieties with enhanced heat stability in terms of AGPase for increasing yields [20,21,22]. In addition, AGPase is also subject to redox-dependent regulation in potato tubers [23], potato leaves, arabidopsis, and peas [24].

Phosphorylation of proteins is involved in various cellular processes and plays a central role in signal transduction [25]. For a protein molecule, phosphorylation can alter enzyme activity, protein conformation and protein–protein interaction, and modulate localization of the protein and its stability [26]. Phosphorylation of proteins can change a protein’s complex formation [27]. In wheat endosperm, phosphorylation of SBEI, SBEIIb and SP can form a protein complex to enhance the synthesis of starch while dephosphorylation of phosphoproteins complex can disturb protein complex formation and reduce the activity of SBEIIa and SBEIIb [28,29]. In the process of starch synthesis, Liu reports that the phosphorylation of SBEI and SBEIIb is involved in heterogenic complexes of proteins [30]. The phosphorylation of SSI and GBSS is detected by phos-tag^TM^ technology [31]. Maize endosperm SBEIIb is phosphorylated on multiple sites [32]. In maize seed development, recent progress in large-scale maize phosphoproteomics has shown that phosphorylation of starch biosynthetic enzymes might play an important role in starch synthesis [33]. However, with AGPase, as a key enzyme in the starch synthetic pathway, its phosphorylation is unclear in the maize kernel starch synthesis process.

Here, we analyzed proteomic data from 08-641 maize endosperm protein enriched by phos-tag^TM^ agarose, which is specific for phosphorylated protein at maize 20 days endosperm lysate after pollination. The proteomic results showed that a total of 1733 proteins were identified from 10,678 distinct peptides with at least one unique peptide. The majority of binding proteins were attributed to the metabolic process and cellular process proteins. A total of 21 unique peptides for Bt2 were identified by mass spectrometry. Bt2 was further demonstrated at 15, 20, and 27 days endosperm by immunoblot. In contrast, Bt2 lost binding when incubated with alkaline phosphatase, which can remove phosphate from protein. Furthermore, the phosphorylation of AGPase subunit Bt2 protein band was further demonstrated by Pro-Q diamond dye staining technology. The phosphorylation sites at Bt2-Ser^10^, Bt2-Thr^451^, and Bt2-Thr^462^ were identified by iTRAQ. In addition, dephosphorylating of AGPase subunit Bt2 decreased its activity in the native gel assay. Taking together, these results suggested that the phosphorylation of AGPase may be a new model for regulating AGPase activity in the starch biosynthesis process.

## 2. Results

### 2.1. Proteomic Analysis of Maize Endosperm Protein Enriched by Phos-tag^TM^ Agarose

**Figure 1:**
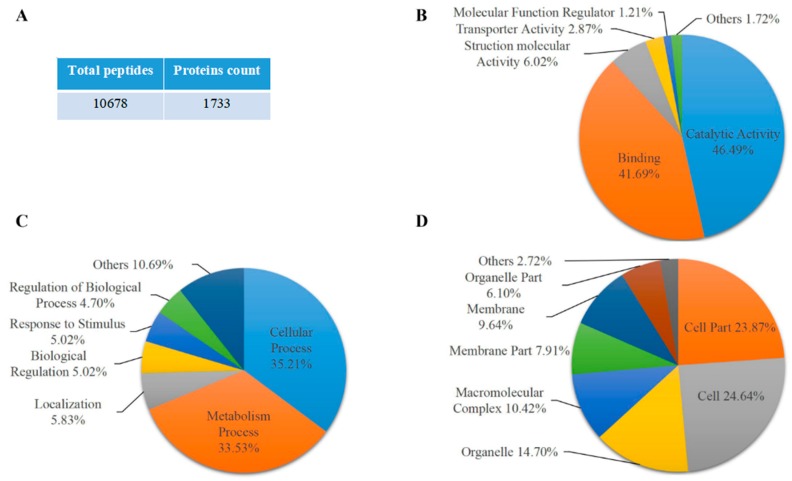

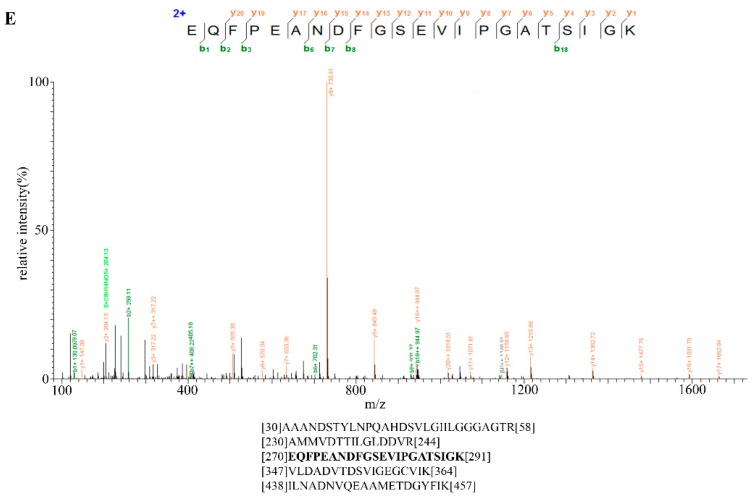
The proteomic analysis of maize endosperm proteins enriched by phos-tag^TM^ agarose. (**A**) Summary of peptides and proteins identified. (**B**) Gene ontology analysis of molecular function level. (**C**) Gene ontology analysis of biological process level. (**D**) Gene ontology analysis of cellular component level. Percentages shown indicated GO term protein accounted for total protein number. (**E**) The representative mass spectrometry diagram and peptides of Bt2 protein. The mass spectrometry diagram shows the bold type of peptide. The numbers show the position of amino acid in Bt2 protein.

To analyze the phosphorylation of protein in maize endosperm at the starch accumulation process, maize endosperm samples were collected at 20 days after pollination from the 08-641 maize inbred line, a high starch content material widely used in southwest China. Maize endosperm protein was extracted from the 08-641 inbred line and then enriched with phos-tag^TM^ agarose that was specific for phosphorylated protein. Protein from phos-tag^TM^ agarose enrichment was performed SDS-PAGE electrophoresis and stained with commassie brilliant blue. The gel was cut for in-gel tryptic digestion for mass spectrometry. The results showed that a total of 1733 proteins were identified from 10,678 peptides with at least one unique peptide (Figure 1A and Table S1). GO term analysis results showed that these proteins identified were annotated into biological process, cellular component and molecular function (Table S2). Of these proteins, 46.49% and 41.69% were annotated into catalytic activity and binding activity, respectively, in the molecular function (Figure 1B). A total of 35.21% and 33.53% of the proteins were cell processes and metabolic processes, respectively, in the biological process (Figure 1C). We also found that 24.64% and 23.87% of the proteins were cell and cell part, respectively (Figure 1D). These results potentially suggested that the phosphorylation of proteins was involved in various biological processes and the molecular function in maize endosperm during the starch synthesis process. Interestingly, the representative peptides of AGPase small subunit Bt2 were identified by mass spectrometry in proteomic data (Figure 1E). The data showed that a total of 21 unique peptides for Bt2 were identified. The cover percentage of peptides for Bt2 was 53.89% (Table S3). Therefore, we speculated that Bt2 might be phosphorylated by kinase; thus, it could be enriched with phos-tag^TM^ agarose.

### 2.2. Antibody Preparation and Evaluation of AGPase Small Subunit Bt2

**Figure 2:**
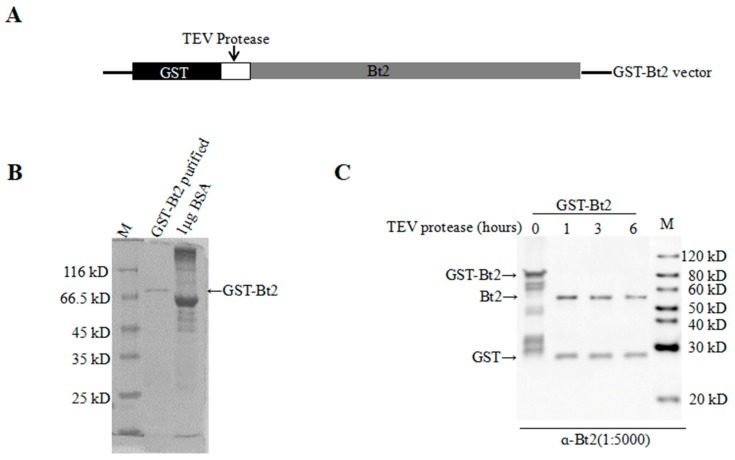
Evaluation of Brittle-2 (Bt2) antibodies using antigen protein purified from GST in vitro. (**A**) Structural diagram of GST-Bt2 vectors. The white region showed the TEV protease site between GST tag and protein interested. (**B**) GST-Bt2 protein purified and BSA standard were detected by coomassie brilliant blue G-250 staining method. The protein marker ladder is indicated in the left column. (**C**) GST-Bt2 digested with TEV protease for 1–6 h by immunoblot. 0.5 μg protein was loaded in each well. Ratio of antibody dilution was 1:5000.

In order to further investigate the phosphorylation of AGPase subunit Bt2, we prepared the antibody Bt2. We purified GST-Bt2 proteins as the antigen for immunization of New Zealand rabbits to prepare the antibody. Through molecule cloning, we successfully constructed pGEX-6-t-GST-Bt2 vectors. The fusion protein could be cut by TEV enzyme between the GST and target proteins (Figure 2A). After induction of protein expression in BL21 bacterial cells with 0.2 mM IPTG, we purified GST-Bt2 proteins with GST beads (Figure 2B). According to the antibody preparation procedure, antiserum was harvested and purified for assessing to detect target protein. In order to evaluate the specificity of antibodies of Bt2, we cut GST-Bt2 proteins with TEV enzyme at different time intervals from 0h to 6 h. Immunoblot results showed Bt2 antibody could recognize Bt2 and GST (Figure 2C). These results showed that Bt2 antiserum could specifically recognize Bt2 proteins in vitro.

### 2.3. Co-Immunoprecipitation of Stromal Protein and Expression Analysis of Bt2 in Maize Endosperm

**Figure 3:**
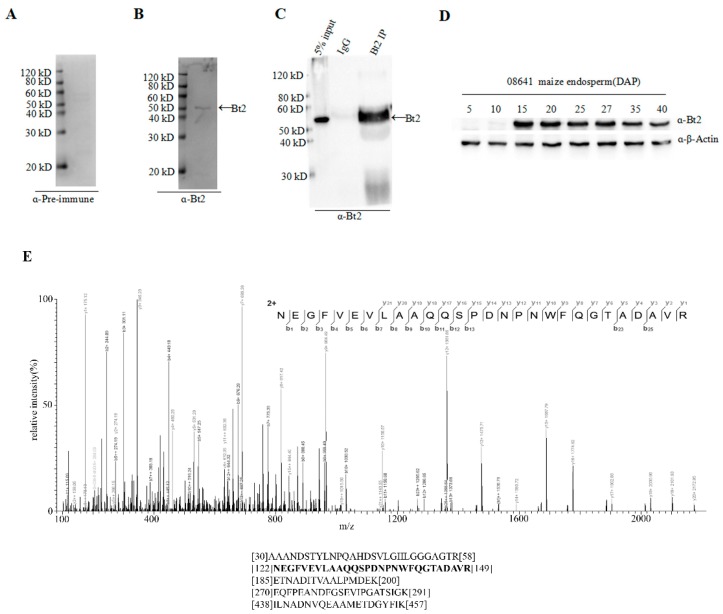
Immunoprecipitation of stromal proteins and expression analysis of Bt2 in maize endosperm. (**A**) Pre-immune serum was evaluated as control with 20 DAP maize endosperm. Ratio of antibodies dilution was 1:5000. (**B**) Bt2 antibody was evaluated by immunoblot with 20 DAP maize endosperm. Ratio of antibodies dilution was 1:5000. Arrow shows Bt2 protein. (**C**) Bt2 antibody was used as immunoprecipitation to detect maize endosperm Bt2 protein by immunoblot. Arrow shows Bt2 protein. (**D**) Expression analysis of Bt2 protein in days indicated after pollination in maize 08-641 endosperm. Ratio of antibodies dilution was 1:5000 for Bt2 and 1:1000 for actin. (**E**) Identification of maize endosperm Bt2 peptides after immunoprecipitation using Bt2 antibody. The protein was precipitated, run the gel, digested with trypsin and analyzed by mass spectrometry. The mass spectrometry diagram showed the bold type of peptide. The numbers show the location of peptides in Bt2 protein.

Specific Bt2 antibody was prepared; however, whether it could recognize Bt2 in maize endosperm in vivo was unclear. In order to further demonstrate the specificity of antibody of Bt2 in vivo, first, we performed immunoblot to detect Bt2 protein from 20 days maize endosperm after pollination with Bt2 antibody (Figure 3B). However, we could not detect Bt2 protein using pre-antiserum (Figure 3A). These results showed that Bt2 antibody was prepared successfully. Second, we used 27 days endosperm after pollination to lysate for immunoprecipitation with Bt2 antibody. The product of immunoprecipitation with Bt2 antibody was identified by immunoblot. In addition, Bt2 protein was immunoprecipited with Bt2 antibody and run the gel and digested with trypsin and analyzed by mass spectrometry. The result showed that Bt2 antibody could immunoprecipite Bt2 protein very specifically and that IgG control could not (Figure 3C). mass spectrometry results showed the band is Bt2 (Figure 3E). Third, we performed the expression analysis of Bt2 proteins in endosperm from 5 days to 40 days after pollination. According to previous reports, the expression of Bt2 is increasing in endosperm from 0 to 20 days after pollination and is decreasing from 20 to 40 days after pollination at mRNA level [34]. We further demonstrated the result by immunoblot using Bt2 antibodies. The result showed that Bt2 protein levels were increasing in endosperm from 0 to 20 days after pollination and then decreased from 25 to 40 days after pollination (Figure 3D). These results showed that Bt2 antibodies could be used for immunoblot, IP assay in vivo and in vitro.

### 2.4. Enrichment of Bt2 Phosphorylation Protein by Phos-tag^TM^

**Figure 4:**
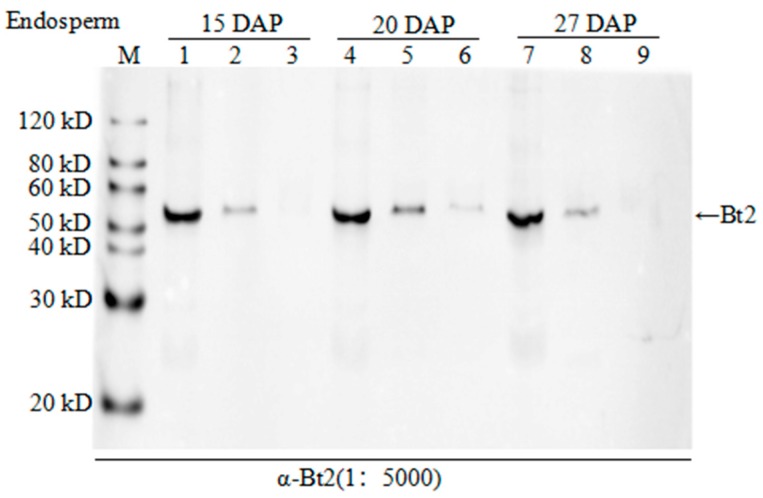
Enrichment of phosphorylated AGPase Bt2. 1, 4, and 7 were 20 µg total protein extracted from maize endosperm 15, 20, and 27 days after pollination, respectively; 2, 5, and 8 were phosphorylated protein enriched from 200 µg total protein by phos-tag^TM^ agarose from maize endosperm 15, 20, and 27 days after pollination, respectively; 3, 6, and 9 were protein from maize endosperm 15, 20, and 27 days after pollination 200 µg total protein treated with alkaline phosphatase and then enriched by phos-tag^TM^ agarose, respectively.

In order to demonstrate the AGPase subunits phosphorylation, we extracted protein from the different development process of endosperm cells in the maize for experiments. After enriching all phosphoproteins from the lysate using phos-tag^TM^ technology and removing nonspecific binding protein by washing, we used the specific Bt2 antibodies to detect Bt2 proteins by immunoblot in phos-tag^TM^ binding protein. Although phos-tag^TM^ enrichment step is hardly get 100% phosphoproteins, we could clearly detect bands of Bt2 protein. However, Bt2 bands would disappear when we added alkaline phosphatase to treat the samples from 15, 20, and 27 days 08-641 maize endosperm after pollination (Figure 4). The result showed that Bt2 might be phosphorylated by kinases in the starch biosythesis process.

### 2.5. Detection of Bt2 Phosphorylation Protein by Diamond Q Staining Technology

**Figure 5:**
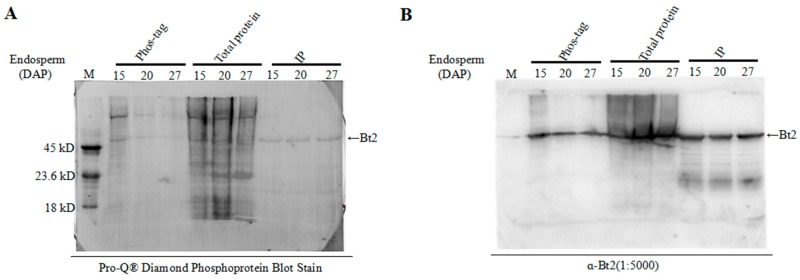
Detection of Bt2 phosphorylation proteins by Diamond Q staining technology. (**A**) Phosphoprotein Pro-Q staining of maize endosperm protein in indicated days after pollination; phos-tag^TM^ group 200 µg total protein was enriched by phos-tag^TM^ agarose with maize endosperm protein; 20 µg total protein was the control, and the IP group 200 µg total protein was immunoprecipitated with the Bt2 antibody; M was a protein marker phosphorylated from Fisher Scientific Company. (**B**) The stained Bt2 protein band was demonstrated by immunoblot. Arrow shows Bt2 band.

Using commercially available phosphorylated proteins as control proteins, phosphorylation-specific staining of maize endosperm proteins was performed to further detect the phosphorylation of Bt2 by Pro-Q diamond dye technology, which is specific for phosphylated proteins [35,36]. Immunoblot was performed to demonstrate Bt2 protein by specific antibody. We harvested maize 15, 20, and 27 days endosperm after pollination and divided it into three groups for assay. The first group was used for enriching phosphoproteins by phos-tag^TM^ technology, the second group was used as control for immunoblot and the third group was used for immunoprecipitation assay using Bt2 antibody. Commercially available phosphorylated proteins were used as a control marker. The results showed that Bt2 was obviously phosphorylated in the maize endosperm, although it appears much loss of phospho-Bt2 through enrichement and transfer-menbrane steps (Figure 5A). Using the specific Bt2 antibody made before, we did a immunoblot test to further demonstrate that the stain band was Bt2 (Figure 5B). These results obviously showed Bt2 was phosphorylated in maize kernel starch biosynthesis.

### 2.6. Identification of Bt2 Phosphorylation Sites by iTRAQ

**Figure 6:**
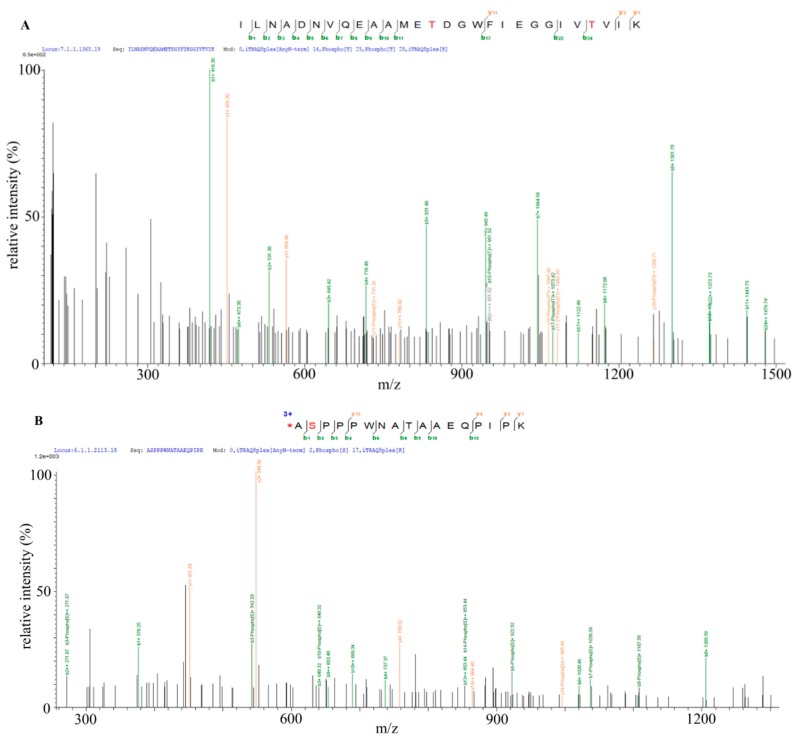

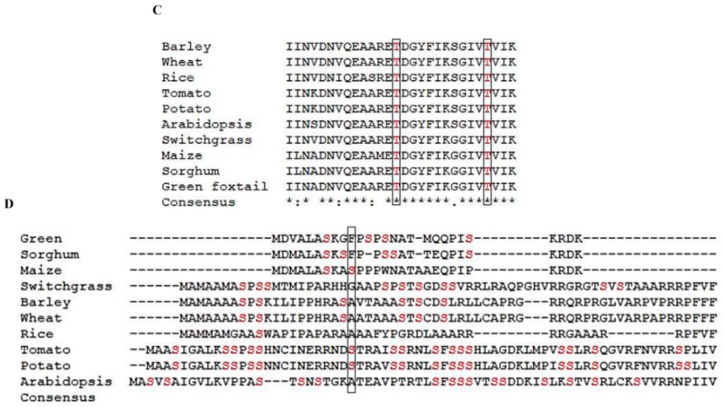
Identification of Bt2 phosphorylation sites by iTRAQ^TM^. (**A**,**B**) Mass spectrometry diagram of Bt2 phosphopeptides. Red letters S and T show the phosphorylated serine and threonine. (**C**,**D**) Multiple alignment analysis of phosphorylated sites of maize Bt2 at Ser^10^, Thr^451^, and Thr^462^ in indicated species by ClustalX 2.1. software. The frame shows the phosphorylated sites identified in maize Bt2 position and putative position in other species. Asterisk shows the consensus of the protein sequence.

The previous results showed that Bt2 was phosphorylated during maize kernel starch biosynthesis. However, the phosphorylation site of Bt2 protein was still unknown. In order to identify the specific phosphorylation sites of Bt2, we collected 08-641 maize 15 DAP endosperm and a transgenic maize to perform iTRAQ assay. The results showed that Bt2-Ser^10^ and Bt2-Thr^451^ and Bt-Thr^462^ were phosphorylated (Figure 6A,B and Table S4). Bioinformatics analysis showed that Bt2-Thr^451^ and Bt2-Thr^462^ were conserved in selected plants (Figure 6C). Instead, Bt2-Ser^10^ was specific for maize, potato, and tomato in 10 selected plants (Figure 6D). These results show that three sites were phosphorylated for Bt2 protein in maize endosperm and that phosphorylation regulation of Bt2 may be complicated in the starch synthesis process.

### 2.7. Enzyme Characteristics of AGPase Phosphorylation and a Potential Regulatory Model

**Figure 7:**
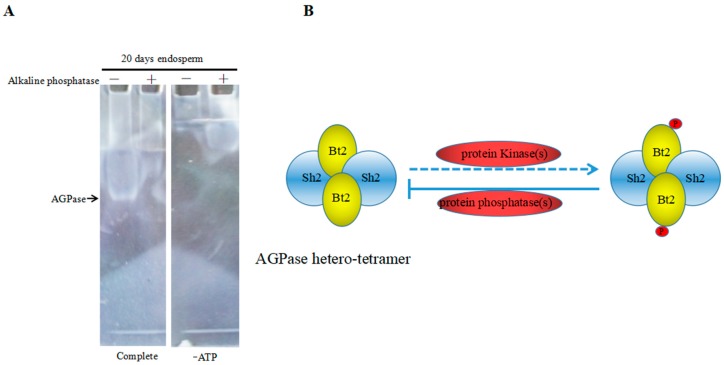
Enzyme characteristics of AGPase dephosphorylation and a potential regulatory model. (**A**) Native gel was performed with 40 µg total maize endosperm protein and protein treated with alkaline phosphatase at 20 days after pollination. Gel was incubated in reaction buffer including ATP and Glc-1-P(Complete) in the presence of divolent cations or in the same buffer lacking ATP (-ATP). Arrow show that the white band is native AGPase band. (**B**) Putative potential regulatory model of phosphorylation of AGPase. Sh2: large subunits.

Previous results suggested that Bt2 could be phosphorylated in maize endosperm during the starch accumulation process. Next, we wanted to know whether the phosphorylation of Bt2 might affect activity of AGPase. We detected phosphorylated AGPase format and dephosphorylated AGPase format by adding alkaline phosphatase to remove the phosphate group. Native gel assay was performed to examine activity of AGPase according to previous reports [34,37]. The active band could be observed in native gel when soaked in the solution, which adds substrate glucose-1-phosphate and ATP in the no-alkaline phosphatase column; however, there are no bands in the alkaline phosphatase or no ATP column (Figure 7A). The results revealed that dephosphorylating of AGPase small subunit Bt2 may abolished or declined the active bands that AGPase catalyzed to form. Therefore, the phosphorylation of Bt2 might be important for AGPase activity. Based on these results, we proposed a potential regulation mode in which phosphorylation of AGPase catalyzed by kinases would enhance its activity. However, dephosphorylation of AGPase catalyzed by alkaline phosphatase would inhibit its activity (Figure 7B). In all, phosphorylation of AGPase subunits may be a new model to regulate the activity of AGPase in the maize kernel starch synthesis process.

## 3. Discussion

AGPase is a key enzyme for catalyzing ATP and Glc-1-p to form ADP-glucose and diphosphate. As the precursor of starch, ADP-glucose is used as a material for incorporating glucosyl units into starch [5]. Genes encoding AGPase activity are mutated in maize, barley, and rice, which leads to a reduction in total endosperm starch in the range of 20–70% of normal. The activity of AGPase is one of determinants for the content of starch in maize kernel. Therefore, it is very important for the regulation of activity of AGPase. To date, there are three main ways reported to regulate the activity of AGPase: small effector molecules for allosteric regulation, thermal regulation and redox regulation [9,10,11,12,38]. In this study, we used phos-tag^TM^ technology, Pro-Q diamond staining and iTRAQ^TM^ to investigate phosphorylation of AGPase. The results show that AGPase subunits Bt2-Ser^10^, Bt2-Thr^451^, and Bt2-Thr^462^ were phosphorylated in the kernel starch accumulation process. In addition, based on the current data, it seems important for the activity of AGPase regulated by phosphorylation. Removal of phosphate groups from AGPase subunits could reduce its activity. Therefore, phosphorylation of AGPase may be an important means of regulating its activity.

The phosphorylation of AGPase leads to binding phos-tag^TM^ agarose. Phos-tag^TM^ agarose is a dinuclear metal complex and acts as a selective phosphate-binding tag molecule specifically for phosphorylated proteins [39,40,41]. According to our current data, phos-tag^TM^ agarose could enrich phosphorylated AGPase, including Bt2. Using SDS to unfold and denature protein, we could still detect Bt2 by phos-tag^TM^ agarose (data not shown). Thus, the result is reliable for the binding of phos-tag^TM^ agarose by the phosphorylation of AGPase. Dephosphorylation of AGPase leads to losing the binding between AGPase subunits and phos-tag^TM^ agarose. Alkaline phosphatase is widely used as a dephosphorylation agent to dephosphorylate various proteins [42,43]. In our experimental system, we used alkaline phosphatase to remove the phosphate group from AGPase, and the binding of phosphorylated AGPase subunit Bt2 with phos-tag^TM^ agarose significantly decreased or disappeared. In this study, we also used a commercially available phosphorylated protein marker as a positive control and Pro-Q diamond staining technology, which is specific for detecting the phosphorylated Bt2 protein in denatured condition by boiling the protein sample. We demonstrated the Bt2 protein by immunoblot using specific Bt2 antibody made in the current experiment. Also, we detected the phosphorylated Bt2 protein. We analyzed AGPase subunit Bt2 peptides mass spectrometry data from 15 DAP maize endosperm by iTRAQ^TM^. Bt2-Ser^10^, Bt2-Thr^451^, and Bt2-Thr^462^ were phosphorylated from mass spectrometry. Therefore, it could be concluded that the phosphorylation of Bt2 actually occurred in vivo from this indirect and direct evidence.

The phosphorylation of AGPase may be complicated and exist in different regulation mechanisms in maize and other species. Walley reported maize AGPase subunit Sh2-Ser^95^ and Bt2-Ser^10^ and Bt2-Ser^104^ were phosphorylated in vivo from proteomic data [33]. Nakagami reported *At*APS1-Thr^231^ and *At*APS1-Thr^236^ were phosphorylated by large-scale comparative phosphoproteomics research in the plant *Arabidopsis thaliana* [44]. Our current data show that Bt2-Ser^10^, Bt2-Thr^451^, and Bt2-Thr^462^ were phosphorylated in maize endosperm. The phopsphorylation of Bt2-Ser^10^ may regulate the activity of AGPase because this site is in N-term of Bt2 [45]. Bt2-Thr^451^ and Bt2-Thr^462^ may affect the structure of AGPase because these sites are in C-term of Bt2 [46,47]. In addition, Bt2-Ser^10^ is a common phosphorylated site for B73 and our inbred maize line. This result suggests that there is common regulatory mechanism in different inbred maize lines. Instead, Bt2-Ser^104^ was phosphorylated in B73, Bt2-Thr^451^ and Bt2-Thr^462^ were phosphorylated in our inbred line. This result also suggests that there are specific regulatory mechanisms in different inbred maize lines. In addition, in different plants, Bt2-Thr^451^ and Bt2-Thr^462^ were very conservative; however, Bt2-Ser^10^ was comparatively specific for maize, potato, and tomato. Thus, it is possible that there are common and specific regulatory mechanisms in different species. Phosphorylation regulation of Bt2 could be complicated in starch synthesis.

The phosphorylation of Bt2 might change the activity and stability of AGPase. In general, phosphorylation of a protein will change the activity or function of enzyme, localization, and binding specificity of target proteins [27]. In order to answer the question of whether it activates or inactivates AGPase after Bt2 phosphorylation, we tried to detect activity of AGPase phosphorylation through native gel assay. Our current results clearly show that the native band disappeared or was non-detectable when alkaline phosphatase was present in the protein sample. We speculate that the result of dephosphorylation of AGPase might inhibit its activity because alkaline phosphatase removing phosphate group from AGPase will lead to a free phosphate group. As previous reported, the free phosphate will inhibit the activity of AGPase [11]. In addition, phosphorylation of SBEI, SBEIIb, and SP is important for stability and activity of the protein complex formed with SBI, SBEI, and SBEIIb. Dephosphorylation of the SBEI-SBEIIb-SP complex will disturb the protein complex and decrease its activity [28,29]. As a heterotetrameric AGPase, which is composed of two identical large Sh2 subunits and two identical small Bt2 subunits, it is potentially possible that like the SBEI-SBEIIb-SP complex, the phosphorylation of AGPase would increase the enzyme stability and activity, the dephosphorylation of AGPase subunits would cause a reduction or loss of its activity and stability.

## 4. Materials and Methods

### 4.1. Plant Materials

Seeds for 08-641 inbred maize line were provided by the maize research institute of Sichuan Agricultural University and grown at the school farm in the summer of 2013–2017. Developing kernels from self-pollinated ears were collected 10 DAP, 15 DAP, 20 DAP, 27 DAP, 30 DAP, 35 DAP, and 40 DAP and were quickly frozen in liquid nitrogen and stored at −80 °C until use. For phos-tag^TM^ enrichment assay, three independent biology repeated maize endosperm samples collected at the same time were mixed as a pool for proteomic analysis.

### 4.2. GST-Gene Fusion System Protein Expression and Purification

GST-gene fusion system protein expression vector pGEX-6t-1-Bt2 were constructed by adding Bt2 genes into the pGEX-6t-1 vector. The cloning primers of Bt2 were as follows: Bt2 Forward: 5′-CG ggatccATGGACTGGCTTTGGCGTCTA-3′, Reverse: 5′-CAGctcgagTCATATAACTGTTCCACTAG GGAG-3′. The lowercase letters indicate the introduced base to create an BamHI and XhoI, respectively. The protein expression and purification of Bt2 were performed according to the GST gene fusion system handbook from GE Healthcare (Piscataway, NY, USA).

### 4.3. Rabbit Breeding, Anti-serum Preparation, and Antibody Purification

New Zealand white rabbits were provided by Da Shuo experimental animal company. Rabbits were maintained in the Animal Core Facility following procedures approved by the Animal Care and Use Committee of Sichuan Agricultural University (no 20160320, Chengdu, China). After one week of acclimation, rabbits were injected with an antigen mixed with adjuvant every two weeks and their venous blood harvested after three injections. Polyclonal rabbit antisera targeted to maize Bt2 were raised against the antigen, in which GST-Bt2 were purified by GST-gene fusion system protein expression assay. Antiserum containing the polyclonal maize antibody was applied to the column with 1 cm^3^ 50% protein A and 50% protein G and washed with 10 cm^3^ ice-cold TBS (50 mM Tris-HCl, pH 7.4, 150 mM NaCl, and 0.05% sodium azide). The antiserum was then thawed in ice water and clarified by centrifugation at 15,000× *g* for 5 min at 4 °C. 3.6 cm^3^ of the clarified antiserum was added to the column and the column was washed with 36 cm^3^ TBS buffer. 2.5 cm^3^ elution buffer with pH 2.7 and pH 1.9 (100 mM glycine pH 2.7 and 100 mM glycine pH 1.9, respectively) was gently added to the column. Roughly 0.4 cm^3^ fractions were collected in the tubes above and neutralized with NB buffer (1 M Tris-HCl, pH 8.0; 1.5 M NaCl; 1 mM EDTA; 0.5% sodium azide) to adjust the pH to approximately 7.4. Pure antibodies were used in immunoblot and immunoprecipitation. Pre-immune sera for each of the antibodies used above were employed as negative controls, and showed no cross-reaction with proteins from maize endosperm lysates and co-immunoprecipitation experiments.

### 4.4. Plant Protein Extraction and Protein Determination

Maize kernels were harvested and quickly frozen in liquid nitrogen and stored at −80 °C until use. Maize endosperm was dissected using pre-chilled tweezers and a mortar on ice, and total endosperm proteins were extracted from endosperm tissues flash frozen in liquid nitrogen. Two grams of endosperm tissue were pulverized using a mortar and pestle under liquid nitrogen. 6 cm^3^ of native protein extraction buffer (100 mM Tris-HCl, pH 7, 10 mM MgCl_2_, 100 mM KCl, 15% glycerol and DDT, 40 mM β-mercaptoethanol, 1 mM PMSF and phosphatase inhibitor cocktail (Sigma) added freshly) were added followed by further shaking in a vortex. The homogenates were centrifuged (16,000× *g*, 10 min, 4 °C) and supernatants stored at −80 °C until use. Protein concentration was determined using the Bio-Rad protein assay (Bio-Rad, Hercules, CA, USA) according to the manufacturer’s instructions and with BSA as a standard.

### 4.5. SDS-PAGE and Immunoblotting

Zn^2+^-phos-tag^TM^ agarose was used to enrich phosphoproteins according to the manufacturer’s instructions (Wako Pure Chemical Industries Ltd., Hiroshima, Japan). Briefly, the total maize endosperm cell lysate sample containing 200 µg protein was used for enriching phosphoproteins with 200 mm^3^ Zn^2+^-phos-tag^TM^ agarose. For dephosphorylation assay, we treated cell lysate by adding alkaline phosphatase. The binding assay was performed for 4 h at 4 °C, and then washed 3 times with washing buffer (0.1 M Tris-CH_3_COOH, 1.0 M CH_3_COONa, pH 7.5). Elution buffer (0.1 M Tris-CH_3_COOH, 1.0 M NaCl, 10 mM NaH_2_PO_4_-NaOH, pH 7.5) was used for the elution of phosphoproteins. Zn^2+^-phos-tag^TM^ agarose binding proteins were separated by electrophoresis. In addition, 20 µg maize endosperm cell lysates were subjected to SDS-PAGE as a control. The SDS polyacrylamide gels consisted of an 8% or 10% acrylamide separation gel and a 4% stacking gel. The resolved proteins were electrophoretically transferred to nitrocellulose membranes (GE Healthcare Life Sciences, Cat: 10600003). The membranes were incubated with anti-Bt2 antibody diluted 1:5000 and anti-actin antibody diluted 1:1000 for 2 h at room temperature. The membranes were then incubated with horseradish peroxidase conjugated with anti-rabbit IgG (Kangwei Company) diluted 1:5000 for 30 min, and the immunoreactive bands were detected using the chemiluminescent substrate, Lumi-Light immunoblotting Substrate (Thermo Scientific, Cat: PI208186 and PJ209602, Rockford, IL, USA). All immunoblot assays were independently performed at least three times.

### 4.6. Mass Spectrometry and Data Processing

The binding protein was collected from phos-tag^TM^ agarose incubated with endosperm protein. Similarly, the production of IP was collected. Protein was performed SDS-PAGE electrophoresis and stained commassie brilliant blue. The gel was cut for in-gel tryptic digestion, gel pieces were destained in 400 mm^3^ 50 mM NH_4_HCO_3_ in 50% acetonitrile (*v*/*v*) until clear. Gel pieces were dehydrated with 100 mm^3^ of 100% acetonitrile for 5 min, the liquid was removed and the gel pieces were rehydrated in 10 mM dithiothreitol and incubated at 56 °C for 60 min. Gel pieces were again dehydrated in 100% acetonitrile, liquid was removed and gel pieces were rehydrated with 55 mM iodoacetamide. Samples were incubated at room temperature in the dark for 45 min. Gel pieces were washed with 50 mM NH_4_HCO_3_ and dehydrated with 100% acetonitrile. Gel pieces were rehydrated with 10 ng/mm^3^ trypsin resuspended in 50 mM NH_4_HCO_3_ on ice for 1 h. Excess liquid was removed and gel pieces were digested with trypsin at 37 °C overnight. Peptides were extracted with 50% acetonitrile and 5% formic acid, followed by 100% acetonitrile. Peptides were dried and re-suspended in 0.1% formic acid.

The tryptic peptides were dissolved in 0.1% formic acid (solvent A), directly loaded onto a homemade reversed-phase analytical column (15 cm in length, 75 μm i.d.). The gradient was comprised of an increase from 4% to 50% solvent B (0.1% formic acid in 98% acetonitrile) in 50 min, 50% to 100% in 4 min, then holding at 100% for the last 6 min, all at a constant flow rate of 400 nl/min on an EASY-nLC 1000 UPLC system. The peptides were subjected to NSI source followed by tandem mass spectrometry (MS/MS) in Q Exactive^TM^ Plus (Thermo) coupled online to the UPLC. The electrospray voltage applied was 2.0 kV. The m/z scan range was 350 to 1800 for full scan, and intact peptides were detected in the Orbitrap at a resolution of 70,000. Peptides were then selected for MS/MS using NCE setting as 28 and the fragments were detected in the Orbitrap at a resolution of 17,500. This was a data-dependent procedure that alternated between one MS scan followed by 20 MS/MS scans with 15.0 s dynamic exclusion. Automatic gain control (AGC) was set at 5E4. The resulting MS/MS data were processed using a Proteome Discoverer 1.3.6 Tandem mass spectra were searched using MASCOT (Matrix Science, London, UK; version 2.2) and against an uniprot Zea mays database (https://www.uniprot.org; 132356 sequences, download on 1 August 2018). Trypsin/P (or other enzymes if any) was specified as the cleavage enzyme, allowing up to 2 missing cleavages. Mass error was set to 10 ppm for precursor ions and 0.02 Da for fragment ions. Peptide confidence was set at high, and the peptide ion score was set >20. The GO term protein function classification in levels 1 and 2 were analyzed by http://www.ebi.ac.uk/GOA/.

### 4.7. Immunoprecipitation (IP) and Co-immunoprecipitation (Co-IP)

Co-immunoprecipitation experiments were conducted using the methods described by Fushan Liu with some modifications [30]. Purified Bt2 antibodies (each approximately 10 µg) were individually used for the immunoprecipitation and co-immunoprecipitation experiments with 27 days endosperm after pollination cell lysates (1 cm^3^, 0.5 mg/cm^3^ proteins). The mixture of antibody and cell lysate was incubated at 4 °C on a rotator for 4 h and precipitation of the antibody performed by adding 20 mm^3^ of Protein A/G-Sepharose (Biorad) made up as a 50% (*w*/*v*) slurry with phosphate buffered saline (PBS, 137 mM NaCl, 10 mM Na_2_HPO_4_, 2.7 mM KCl, 1.8 mM KH_2_PO_4_, pH 7.4) at 4 °C for 4 h. The Protein A-Sepharose/antibody/protein complex was centrifuged at 2000× *g* for 5 min at 4 °C in a refrigerated microfuge and the supernatant was discarded. The pellet was washed five times (1.0 cm^3^ each) with PBS, followed by washing five times with a buffer containing 10 mM HEPES-NaOH, pH 7.5, and 150 mM NaCl. Washed pellets were boiled in SDS loading buffer and separated by SDS-PAGE, followed by immunoblot analysis.

### 4.8. Pro-Q Diamond Phosphoprotein Staining

For the control group, 20 µg total protein were extracted from 08-641 maize endosperm 15 days, 20 days and 27 days after pollination for running gel. For the phos-tag^TM^ group, a total of 200 µg maize endosperm cell lysate protein was enriched with 20 mm^3^ slurry made up as Zn^2+^-phos-tag^TM^ agarose and a 50% (*v*/*v*) suspension buffer containing 20 mM Tris-CH_3_COOH pH 7.4, 20% (*v*/*v*) 3-propanol. For the immunoprecipitation (IP) group, 200 µg protein from endosperm was incubated with the control IgG and Bt2, respectively. Then precipitation of the antibody was performed by adding 20 mm^3^ of Protein A/G-Sepharose (Biorad) made up as a 50% (*w*/*v*) slurry with phosphate buffered saline (PBS, 137 mM NaCl, 10 mM Na_2_HPO_4_, 2.7 mM KCl, 1.8 mM KH_2_PO_4_, pH 7.4) at 4 °C on a rotator for 4 h. The Protein A-Sepharose/antibody/protein complex was centrifuged at 2000× *g* for 5 min at 4 °C in a refrigerated microfuge, and the supernatant was discarded. The pellet was washed five times (1.0 cm^3^ each) with PBS buffer. Washed pellets were boiled in SDS loading buffer and separated by SDS-PAGE. For all protein samples, transferring protein to a polyvinylidene difluoride (PVDF) membrane (GE Healthcare Life Sciences, Cat: 10600023) was used for Pro-Q diamond phosphoprotein dye staining according to manufacturer’s instruction from Invitrogen (Cat: MP33300). A commercially available phosphorylated protein marker was used as a positive control and was purchased from Thermo Fisher Scientific (Cat: MP33350). In order to exclude the possibility that the signal of Pro-Q diamond phosphoprotein dye interfered with the signal from immunoblotting, after destaining of the PVDF membrane with Pro-Q diamond phosphoprotein dye, the PVDF membrane with the protein sample was used for Bt2 immunoblotting.

### 4.9. iTRAQ^TM^ Labeling and Mass Spectrometry Analysis

Phosphorylated protein was identified by the iTRAQ^TM^ method described by and Ma [48]. Briefly, three biological repeats maize 15 DAP kernels and three dependent transgene samples protein were extracted and digested by sequencing-grade trypsin. Peptides were collected and lyophilized. The samples were reconstituted in TEAB buffer and three independent samples labelled by iTRAQ reagents (8 PLEX multiplex kit, AB Sciex, Cat: 4381663). Three independent WT samples were respectively labeled with iTRAQ tag 115,116,117. Three independent transgenic samples were respectively labeled with iTRAQ tag 118,119,121. TiO_2_ was used to enrich the phosphopeptides for mass spectrometry analysis. All analyses were performed by a Triple TOF 5600 Mass Spectrometer (SCIEX, USA) equipped with a Nanospray III source (SCIEX, Framingham, MA, USA). Samples were loaded by a capillary C18 trap column (3 cm × 100 µm) and then separated by a C18 column (15 cm × 75 µm) on an Eksigent nanoLC-1D plus system (SCIEX, USA). The flow rate was 300 nL/min and the linear gradient was 90 min (from 5–85% B over 67 min; mobile phase A = 2%ACN/0.1%FA and B = 95%ACN/0.1%FA). Data were acquired with a 2.4 kV ion spray voltage, 35 psi curtain gas, 5 psi nebulizer gas, and an interface heater temperature of 150 °C. The MS scanned between 400 and 1500 with an accumulation time of 250 ms. For IDA, 30 MS/MS spectra (80 ms each, mass range 100–1500) were acquired with MS peaks above intensity 260 and a charge state of between 2 and 5. A rolling collision energy voltage was used for CID fragmentation for MS/MS spectra acquisitions. Mass was dynamically excluded for 22 s.

### 4.10. Zymograms of Native PAGE

AGPase zymograms modified method was used for in-gel assay of AGPase activity as described by Huang et al. [34]. Five endosperm samples were ground in liquid nitrogen using a pre-chilled mortar and pestle, and an extraction buffer (100 mM Tris-HCl, pH 7, 40 mM β-mercaptoethanol added freshly, 10 mM MgCl_2_, 100 mM KCl, and 15% glycerol) was added in 1.5 cm^3^ EP tubes. The homogenate was centrifuged at 16,000× *g* for 30 min at 4°C, and the supernatant was stored at −80 °C or used immediately. Equal amounts of protein (40 µg) were loaded onto a 7.5% (*w*/*v*) native acrylamide gel and electrophoresed in Laemmli buffer lacking SDS at 4 °C at 90 V for 2 to 4 h. The gel was then incubated overnight at 37 °C in 100 mM Tris-HCl, pH 8, 5 mM β-mercaptoethanol, 5 mM CaCl_2_, 10 mM MgCl_2_, 5 mM Glc-1-P, 5 mM ATP, and 10 mM 3-PGA. Gels were photographed on a dark background to visualize white precipitate bands. Control assays omitted ATP.

## 5. Conclusions

We provide the evidence that AGPase subunit Bt2 was phosphorylated in the maize endosperm during starch synthesis process. Proteomic and mass spectrometry analysis showed the peptides of AGPase subunit Bt2 were identified from product of phos-tag^TM^ agarose binding maize endosperm proteins. Phosphorylation of AGPase subunits could bind phos-tag^TM^ agarose. However, dephosphorylation of AGPase subunits would lose the binding treating with ALP. We further demonstrated the result by immunoblot through specific Bt2 antibodies. Pro-Q diamond staining further demonstrated the phosphorylation of Bt2. The specific phosphorylation sites of Bt2 at Ser^10^, Thr^451^, and Thr^462^ were identified by iTRAQ^TM^. Protein sequence multiple alignment analysis showed Bt2-Thr^451^ and Bt2-Thr^462^ were very conserved sites in different species. Bt2-Ser^10^ is specific for maize, potato and tomato. In native gel assay, removing phosphate group from Bt2 with alkaline phosphatase, the activity of AGPase was abolished. In all, our data potentially suggest that phosphorylation of Bt2 may be a new model to regulate activity of AGPase.

## Supplementary Materials

Supplementary materials can be found at https://www.mdpi.com/1422-0067/20/4/986/s1.

## Abbreviations

ALP	Alkaline phosphatase
Co-IP	Co-immunoprecipitation
DAP	Days After Pollination
GBSS	Granule bound starch synthase
IP	immunoprecipitation
SBEI	Starch Branch Enzyme I
SBEIIa	Starch Branch Enzyme IIa
SP	Starch Phosphorylase
SSI	Starch synthase I
SSII	Starch synthase II
